# Comparative analysis of the *Photorhabdus luminescens *and the *Yersinia enterocolitica *genomes: uncovering candidate genes involved in insect pathogenicity

**DOI:** 10.1186/1471-2164-9-40

**Published:** 2008-01-25

**Authors:** Ralf Heermann, Thilo M Fuchs

**Affiliations:** 1 Ludwig-Maximilians-Universität München, Department Biologie I, Bereich Mikrobiologie, Maria-Ward-Str. 1a, D-80638 München, Germany; 2 Zentralinstitut für Ernährungs- und Lebensmittelforschung (ZIEL), Abteilung Mikrobiologie, Technische Universität München, Weihenstephaner Berg 3, D-85354 Freising, Germany

## Abstract

**Background:**

*Photorhabdus luminescens *and *Yersinia enterocolitica *are both enteric bacteria which are associated with insects. *P. luminescens *lives in symbiosis with soil nematodes and is highly pathogenic towards insects but not to humans. In contrast, *Y. enterocolitica *is widely found in the environment and mainly known to cause gastroenteritis in men, but has only recently been shown to be also toxic for insects. It is expected that both pathogens share an overlap of genetic determinants that play a role within the insect host.

**Results:**

A selective genome comparison was applied. Proteins belonging to the class of two-component regulatory systems, quorum sensing, universal stress proteins, and c-di-GMP signalling have been analysed. The interorganismic synopsis of selected regulatory systems uncovered common and distinct signalling mechanisms of both pathogens used for perception of signals within the insect host. Particularly, a new class of LuxR-like regulators was identified, which might be involved in detecting insect-specific molecules. In addition, the genetic overlap unravelled a two-component system that is unique for the genera *Photorhabdus *and *Yersinia *and is therefore suggested to play a major role in the pathogen-insect relationship. Our analysis also highlights factors of both pathogens that are expressed at low temperatures as encountered in insects in contrast to higher (body) temperature, providing evidence that temperature is a yet under-investigated environmental signal for bacterial adaptation to various hosts. Common degradative metabolic pathways are described that might be used to explore nutrients within the insect gut or hemolymph, thus enabling the proliferation of *P. luminescens *and *Y. enterocolitica *in their invertebrate hosts. A strikingly higher number of genes encoding insecticidal toxins and other virulence factors in *P. luminescens *compared to *Y. enterocolitica *correlates with the higher virulence of *P. luminescens *towards insects, and suggests a putative broader insect host spectrum of this pathogen.

**Conclusion:**

A set of factors shared by the two pathogens was identified including those that are involved in the host infection process, in persistence within the insect, or in host exploitation. Some of them might have been selected during the association with insects and then adapted to pathogenesis in mammalian hosts.

## Background

Pathogenicity as well as symbiosis plays a key role in the interaction of bacteria with their hosts including invertebrates. Despite the relevance of this relationship for the evolution of bacterial pathogenicity, few studies have addressed this subject at the genomic level. We therefore decided to perform a comparative study of the genomes of *Photorhabdus luminescens *and *Yersinia enterocolitica*. The former bacterium is a representative of pathogens highly virulent towards insects, but apathogenic against men. *Y. enterocolitica*, an example of a primarily human pathogen, also confers toxicity to insects, but is less toxic towards these hosts than *P. luminescens*.

Members of the genus *Yersinia *are primarily considered as mammalian pathogens. However, *Y. pestis*, a blood-borne pathogen and the etiological agent of human plague, has long been known to be transmitted by insects, specifically by rat fleas. *Y. enterocolitica *strains have been isolated from flies that are assumed to play an important role in food contamination by this pathogen [1-3], and *Y. pseudotuberculosis *strains were recovered from fly larvae isolated in the wild [4]. More recent data strongly support the idea that yersiniae are capable to interact with insects. Loci encoding the insecticidal toxin complexes (Tc) have been identified in the genomes of *Y. pestis *KIM [5], *Y. pseudotuberculosis *[6], and *Y. enterocolitica *[7]. *Y. pseudotuberculosis*, in contrast to *Y. pestis*, has been shown to be orally toxic to flea [8]. This toxicity revealed to be independent of *tc *genes, suggesting that loss of one or more insect gut toxins is a critical step in the change of the *Y. pestis *lifestyle compared with the *Y. pseudotuberculosis *and thus in evolution of flea-borne transmission [8]. While *Y. enterocolitica *and *Y. pseudotuberculosis *have diverged within the last 200 million years, *Y. pestis *has emerged from *Y. pseudotuberculosis *only 1,500–20,000 years ago [9]. Bacterial lysates both of *Y. enterocolitica *and *Y. pseudotuberculosis *are toxic for *Manduca sexta *neonates, and significant levels of natively or heterologously expressed toxins were observed in both species at 15°C, but not at mammalian body temperature [7,10]. Furthermore, *Y. pseudotuberculosis *and *Y. enterocolitica *have been demonstrated to adhere to and invade cultivated insect cells [10]. Thus, the interaction of *Y. enterocolitica *with insects is an important link in the ecological range of bacteria-host interactions extending from entomopathogenic to humanpathogenic bacteria.

**Figure 1:**
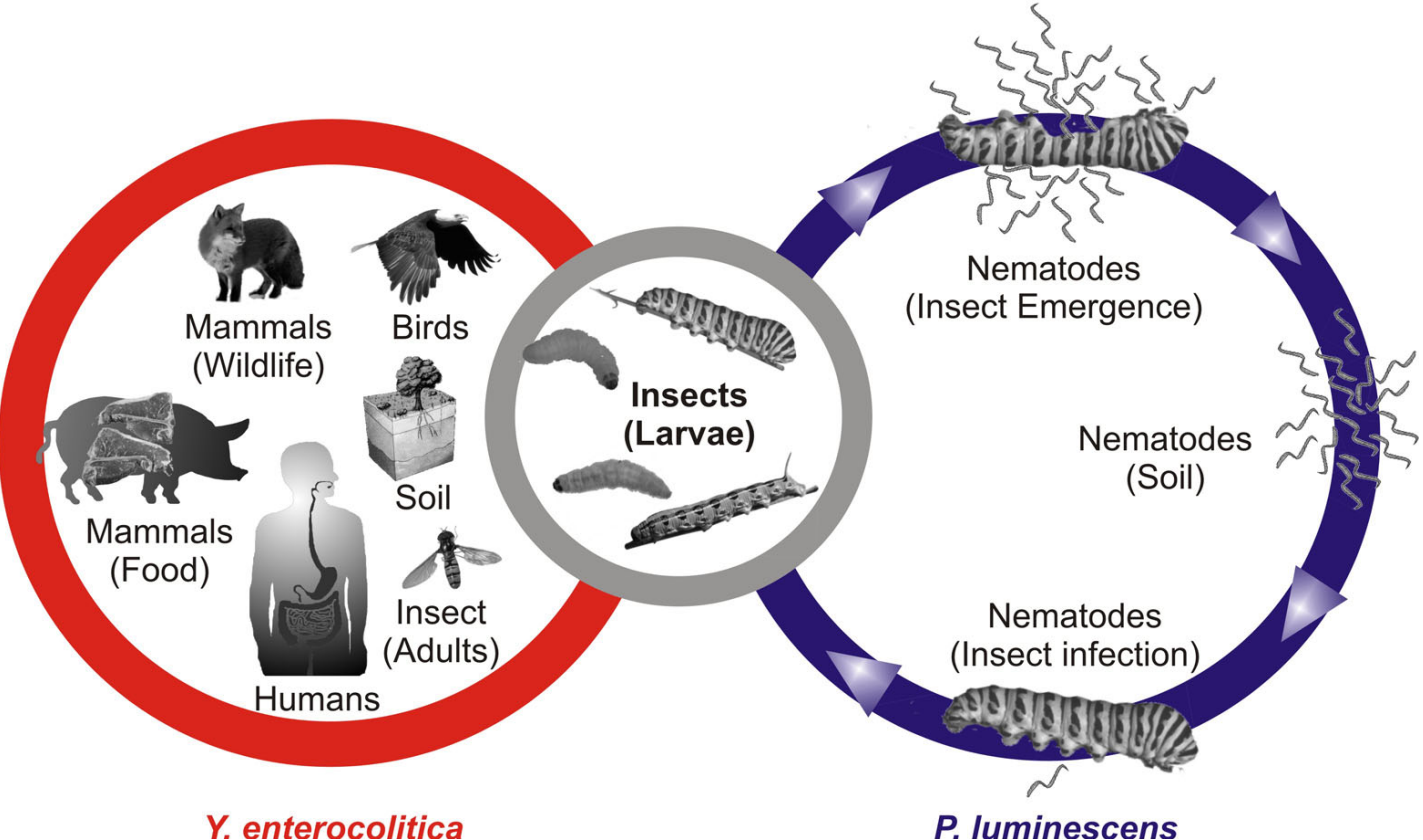
**The life cycles of *P. luminescens *and *Y. enterocolitica***. Right: *P. luminescens *is an endosymbiont of the nematode species *H. bacteriophora*, both living in a highly specific symbiosis. When the nematodes once have infected the insect larvae, they release the highly entomopathogenic bacteria directly into the hemocoel, resulting in a rapid death of the host. The carcass is a rich food source allowing proliferation of both the nematodes and the bacteria. When the cadaver is depleted, nematodes and bacteria reassociate, emerge from the insect, and scan the soil for new victims. Left: *Y. enterocolitica *is found in the soil, in water, in meat or within the gastrointestinal tract of birds [130] or mammals, but is primarily considered as a human pathogen. Middle: *Y. enterocolitica *are able to infect mammals, but are also toxic to insects which are assumed to play a role in evolution and transmission of this bacterium. In contrast to *P. luminescens *which is infectious only towards insect larvae, *Y. enterocolitica *has also been isolated from adult insects [1]. The life cycle stage shared by *P. luminescens *and *Y. enterocolitica *corresponds to a common pool of virulence factors as shown by genome dissection presented here.

In contrast, *Photorhabdus luminescens *is predominantly an insect pathogenic enterobacterium which maintains a mutualistic interaction with heterorhabditid nematodes, and can infect a wide range of insects [11,12]. Interestingly, another *Photorhabdus *species, *P. asymbiotica*, has been described as a human pathogen. It was isolated from human clinical specimens where the cells caused locally invasive soft tissue infections [13,14]. It is assumed that these strains are associated with spiders, because spider bites where attended with *Photorhabdus *human infections [15]. However, bacteria of the species *P. luminescens *are exclusively known to be associated with nematodes and insects. Generally, the bacteria colonise the gut of the infective juvenile stage of the nematode *Heterorhabditis bacteriophora*. Upon entering an insect host, the nematodes release the bacteria by regurgiation directly into the insect hemocoel, the open circulatory system of the insect. Once inside the hemocoel, the bacteria replicate rapidly and establish a lethal septemica in the host by the production of virulence factors such as the insecticidal toxin complexes that kill the insect within 48 hours. Bioconversion of the insect's body by *P. luminescens *produces a rich food source for the bacteria as well as for the nematodes. Nematode reproduction is supported by the bacteria, probably by providing essential nutrients that are required for efficient nematode proliferation [16]. Further properties of *P. luminescens *are the production of many antimicrobial substances to defend the insect cadaver from bacterial competitors, and glowing due to bacterial luciferase production. When the insect cadaver is depleted, the nematodes and bacteria reassociate and emerge from the carcass in search of a new insect host (Fig. 1, right circle)[17,18]. *Photorhabdus *species exist in two forms, designated as primary and secondary phenotypic colony variants, which differ in morphological and physiological traits. Primary variants are found to produce extracellular protease, extracellular lipase, intracellular protein crystals CipA and CipB, antibiotics, and are bioluminescent. Secondary variants lack protease, lipase and antibiotic activity, and bioluminescence is strongly decreased. They also differ in colony morphology, pigmentation, dye adsorption, metabolism, and the ability to support growth and reproduction of the nematodes. It is assumed that primary variants correspond to the nematode-associated form, and secondary variants to the insect-associated form of the bacteria [19,20]. Therefore, *P. luminescens *serves as an ideal model to study the switch from a symbiotic state with nematodes to one in which the bacterium is pathogenic to insects [21,22].

In the following comparative genome analysis, we examined the extent to which *P. luminescens *and *Y. enterocolitica *share factors that are probably attributed to insect association. We identified genes and the corresponding proteins involved in signalling, regulation, pathogenicity, as well as in metabolism, and suggest their possible function during colonization and infection of non-mammals. The results obtained not only improve our understanding of the biology of both pathogens, but also reveal some implications on the evolution of invertebrate and vertebrate virulence factors.

## Results and Discussion

The genomes of *P. luminescens *ssp. *laumondii *TT01 and *Y. enterocolitica *8081 have completely been sequenced. The genome of the latter strain has a size of ~4.6 Mbp and encodes 4037 putative proteins [23]. Its genome size is exceeded by the ~5.7 Mbp genome of *P. luminescens *encoding 4839 putative proteins [24]. To uncover candidate genes which are involved in insect pathogenicity, a total of 424 (*P. luminescens*) and 386 (*Y. enterocolitica*) genes and proteins predicted to belong to one of the functional categories described in the text were analysed for their presence or absence in both organisms, and for their degree of similarity. House-keeping genes and genes of unknown function were not considered. The set of shared genes or proteins, respectively, indicates mechanisms of regulation, virulence and metabolic pathways similar for both pathogens, and moreover unraveled novel candidate genes/proteins which presumably are involved in insect association and/or pathogenicity. Proteins which are solely present in either one of the organisms suggest a distinct function of these factors, or different strategies followed by the two pathogens during their life cycles.

### Sensing, signalling, and regulation

Bacteria have evolved several regulation mechanisms to ensure a proper answer to changing environments. Upon entering their insect hosts, *P. luminescens *and *Y. enterocolitica *are challenged by varying and detrimental surrounding conditions which they have to sense and adapt to for further persistence. In addition, both pathogens must be capable to withstand the insect's immune response. In the following chapter we compare sensing and regulating mechanisms of the two insect-associated organisms, *P. luminescens and Y. enterocolitica*, thus identifying strategies which might be important for insect colonization and pathogenicity.

#### Two-component signal transduction

**Figure 2:**
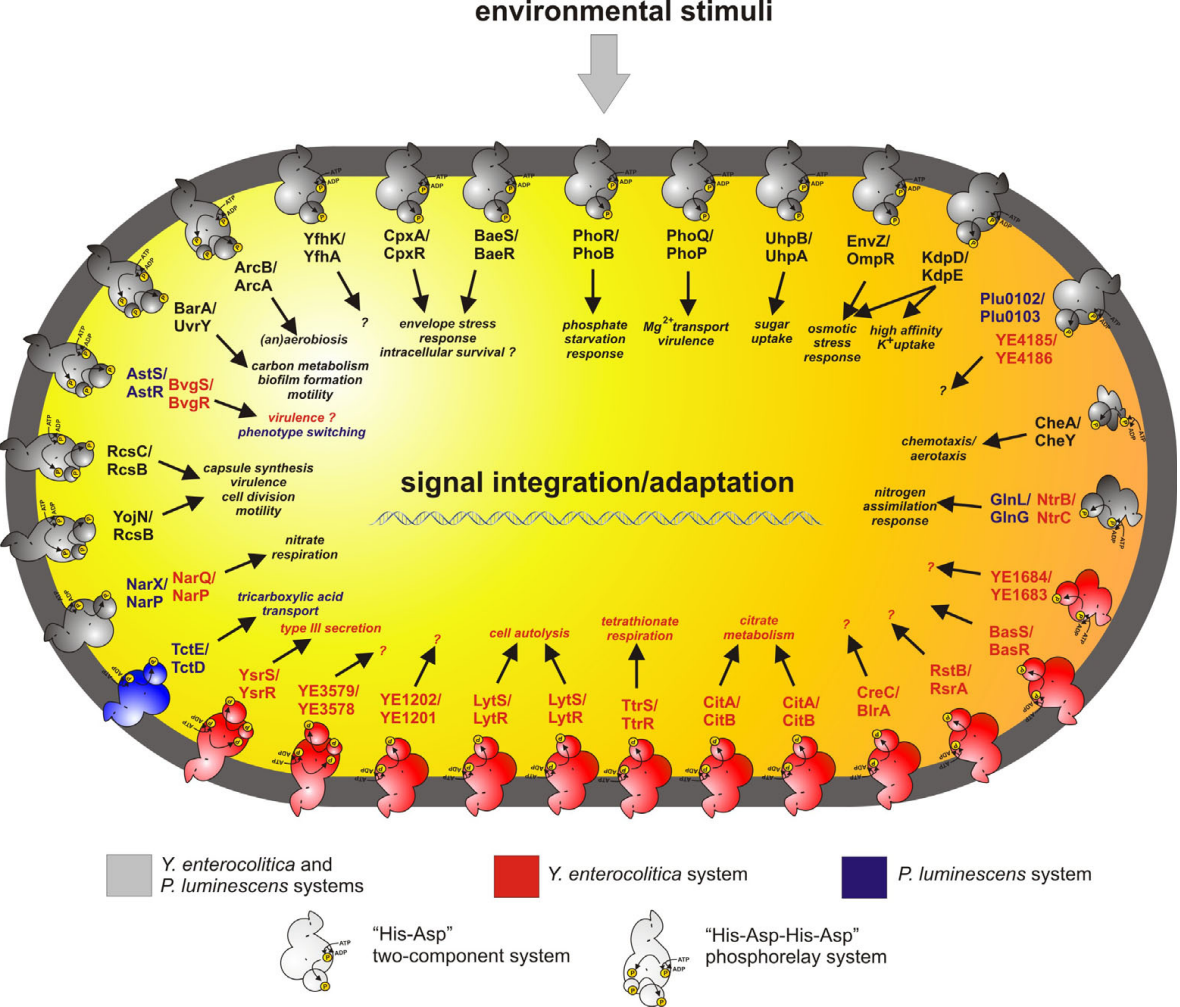
**Two-component systems in *P. luminescens *and *Y. enterocolitica***. 18 TCSs are present in *P. luminescens*, and 28 TCSs in *Y. enterocolitica*. Both organisms share 17 of these systems (grey colour). One is singular to *P. luminescens *(coloured in blue), but 11 to *Y. enterocolitica *(red colour). Basic TCSs (His-Asp phosphotransfer) and complex phosphorelay systems (His-Asp-His-Asp phosphorelay) are distinguished by different drawings. The 17 systems shared by the two pathogens, and also present in other (enteric) bacteria, are CpxAR and BaeSR (Envelope stress, [131]), CheAYW (motility, [132]), PhoRB (Phosphate starvation, [133]), UhpBA (Sugar uptake, [134]), ArcBA (aerob/anaerob respiration, [135]), BarA/UvrY (carbon metabolism, motility, biofilm formation, [136]), RcsC/RcsD (capsular synthesis, virulence, [137]), KdpD/KdpE (K^+^-limitation, osmotic stress, [138]), EnvZ/OmpR (osmotic stress, [139]), NtrB/NtrC and GlnL/GlnG (nitrogen assimilation, [140]), PhoQ/PhoP (Mg^2+ ^sensing, virulence, [141]), BvgSR/AstSR (virulence, phenotypic switching, [28, 142]), RcsC/RcsB and YojN/RcsB (capsule synthesis, cell division, motility, virulence, [143, 144]), and YfhK/YfhA, a system of unknown function. Furthermore, a TCS exists in both organisms which is unique for the genera *Photorhabdus *and *Yersinia *and cannot be found with a comparable homology/identity degree in any other yet known organism (Plu0102/Plu0103 and YE4185/YE4186). The 11 systems which are present in *Y. enterocolitica*, but not in *P. luminescens*, are YsrS/YsrR (activation of a *Yersinia *specific type-III secretion system, [87]), LytS/LytR (cell autolysis, [145]), CitA/CitB (citrate metabolism, [146]), TtrS/TtrR (tetrathionate respiration, [99]), and six systems of unknown function (YE3579/YE3578, YE1202/YE1201, YE1684/YE1683, CreC/BlrA, RstB/RstA, and BasS/BasR).

To sense their environment and to react rapidly to changing surrounding conditions, bacteria have evolved so called two-component systems (TCSs) [25] which have been found to be involved in the control of virulence or symbiosis, in metabolite utilization, and also in the adaptation to various stress factors [26]. A basic TCS consists of two proteins, a sensor histidine kinase and a response regulator performing a His-Asp phosphotransfer. The consisting domains or proteins can also be organized as more complex systems using a His-Asp-His-Asp phosphorelay. The number of TCSs ranges from zero in *Mycoplasma genitalium *to 80 in *Syncheocystes *spp. [25,27]. Eighteen of these TCSs are present in *P. luminescens*, and 28 in *Y. enterocolitica*, of which 17 are shared by both organisms (Fig. 2, depicted in grey). The additional set of eleven TCSs in *Y. enterocolitica *(Fig. 2, shown in red) might reflect the high number of different environments this pathogen is exposed to during its life cycle, namely soil, water and invertebrates as well as mammalian hosts. In contrast, *P. luminescens *cells are primarily restricted to symbiosis with the nematode species *H. bacteriophora *and the insect larvae as hosts, thus encountering a more homeostatic milieu. Among the eleven TCSs of *Y. enterocolitica *not shared by *P. luminescens *are duplicates of the CitA/CitB system (YE2505/YE2506 and YE2654/YE2655) and of the LytS/LytR system (YE1228/YE1227 and YE4014/YE4015). The principal biological reason for this redundancy remains unclear. Interestingly, one TCS (Plu0102/Plu103 and YE4185/YE4186) is unique for the genera *Photorhabdus *and *Yersinia*. Both sensor kinases Plu0102 and YE4185 are of moderate similarity (31.5% identity, 48.5% homology). They are anchored to the membrane with one transmembrane domain, and have a large periplasmic sensing domain which is proposed to bind a specific ligand. Therefore, Plu0102 and YE4185 are interesting candidates for unravelling invertebrate-specific signals. The putative target genes of Plu0102/Plu0103 and Ye4185/Ye4186, *plu0104 *and *ye4187*, respectively, are homologues and encode putative secreted proteins which might act in a similar, yet unknown manner.

All TCSs present in both organisms are depicted in grey in Fig. 2, and include PhoP/PhoQ, and AstS/AstR (BvgS/BvgR) which have been identified to be involved in virulence [28]. The role of PhoP/PhoQ in regulating virulence gene expression has been characterized mainly in *Salmonella *species, but has also been shown, in addition to three other TCSs, to be important for virulence of *Y. pseudotuberculosis *[29,30]. In *P. luminescens*, this TCS controls the expression of the *pbgPE *operon which is involved in lipid A modification and thus plays a role in colonization and infection of the invertebrate hosts [18,31]. Furthermore, PhoP has also been found to be important for virulence of *Y. pestis *[32], but its function in *Y. enterocolitica *during its insect-associated phase remains hypothetical. The AstS/AstR TCS is required for the correct timing of phase variant switching in *P. luminescens *[28]. BvgS/BvgR is the TCS of *Y. enterocolitica *that corresponds to AstS/AstR. Because *Y. enterocolitica *is not known to switch to another phenotypic variant, the possible role in virulence regulation still remains to be elucidated. Both *Y. enterocolitica *and *P. luminescens *produce the KdpD/KdpE system that regulates K^+ ^homeostasis and osmotic stress. It has recently been found that the Kdp-system of *P. luminescens *is important for insect pathogenicity (S. E. Reynolds and N. R. Waterfield, University of Bath, UK, personal communication). Therefore, the KdpD/KdpE system is also a further candidate system which might be involved in the regulation of insecticidal activity of *Y. enterocolitica*.

The only TCS of *P. luminescens *absent in *Y. enterocolitica *is TctE/TctD (Fig. 2, marked in blue), which, however, is found in the genomes of *Y. intermedia *and *Y. frederiksenii*. Beside these microorganisms, TctE/TctD homologues controlling the transport of tricarboxylic acid (see section "Tricarboxylate utilization") are present in the genera *Salmonella*, *Burkholderia*, *Agrobacterium*, *Bordetella*, *Collinsella*, *Xylella*, *Xanthomonas*, and *Pseudomonas*, particularly *P. entomophila*, all of which are found in association with eukaryotes.

To summarize, the comparison of the *P. luminescens *and the *Y. enterocolitica *TCSs reveals a basal set of signal sensing mechanisms which are used by both organisms. Whether the stimulons or regulons which are regulated by these sets of TCSs are also similar remains to be examined. In comparison to *P. luminescens, Y. enterocolitica *uses an expanded set of TCSs, possibly to adapt to its various hosts (Fig. 1).

#### Quorum sensing-like gene regulation

##### Regulation by AHL-LuxR-like receptors

**Figure 3:**
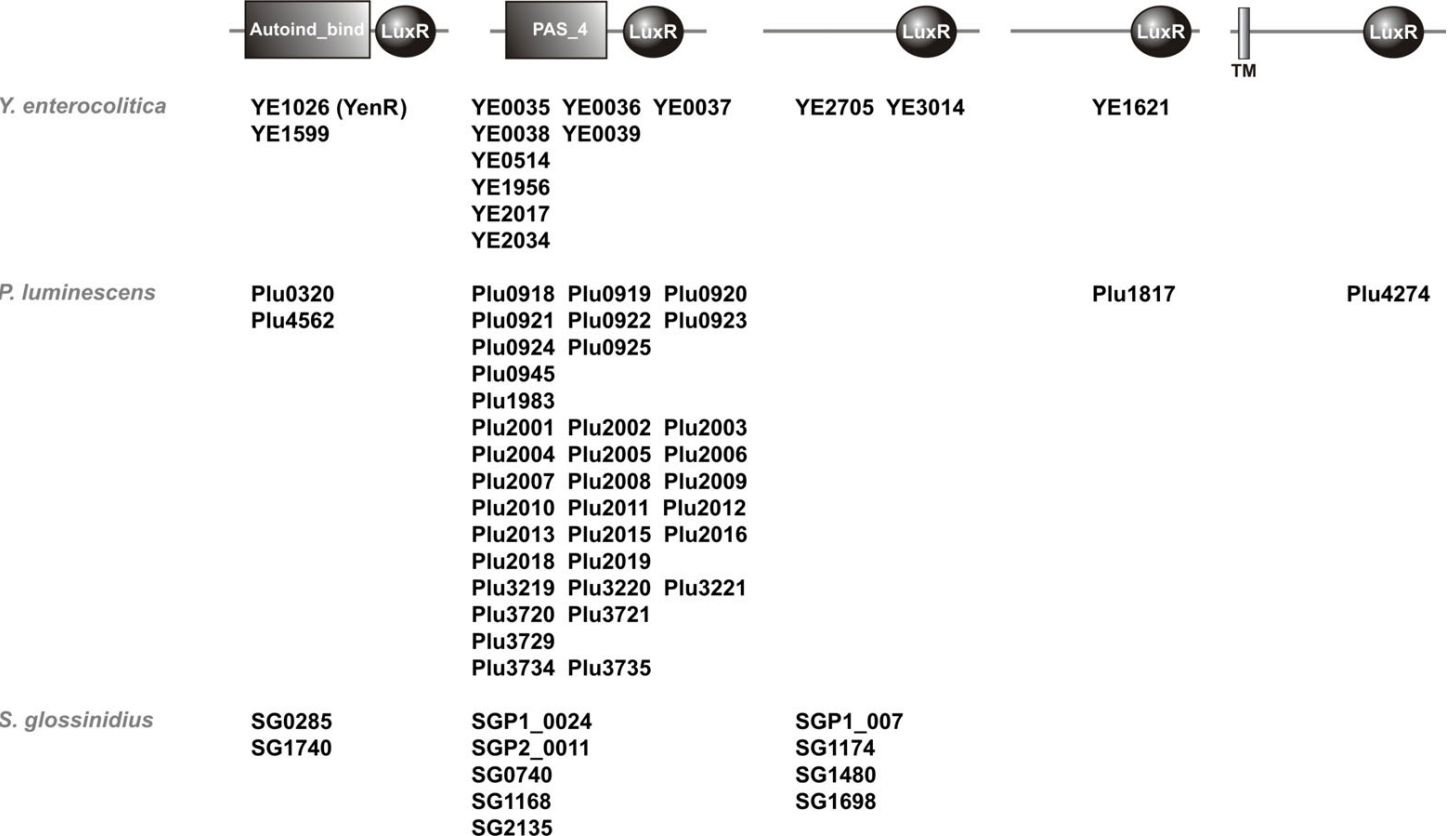
**LuxR-like receptors in *P. luminescens*, *Y. enterocolitica*, and *S. glossinidius***. The five types of different LuxR-like receptors and their homologues in these three organisms are shown (see text for details). The "HTH_LuxR" motif (SMART00421) is indicated by a circle, the "autoinductor-binding"-domain (PFAM03472) and the "PAS_4"-domain (PFAM08448) by boxes. TM: transmembrane domain.

Virulence, bioluminescence, mutualism, antibiotic production and biofilm formation are often regulated by LuxI/LuxR quorum sensing systems in Gram-negative bacteria. They produce membrane diffusible signalling molecules, acyl homoserine lactones (AHLs), which are sensed by the receptor/regulator LuxR when exceeding a threshold concentration. These AHLs are produced by an autoinductor synthase named LuxI. Upon autoinductor-binding, the receptor LuxR binds to the promoter/operator regions of the target genes or operons, resulting in the regulation of gene expression in response to the cell number [33]. *Y. enterocolitica *possesses a typical quorum sensing pair of homologues, YenI/YenR (YE1600/YE1599), and it has recently been shown that swimming and swarming motility is regulated by 3-oxo-C6-AHL and C6-AHL, which are synthesized by YenI [34]. In *Y. pestis*, the production of YspI and YspR, the homologues of YenI and YenR, is induced at 26°C (Table 1). Moreover, we identified a second AHL-LuxR regulator, YE1026, which lacks a separate AHL synthase (Fig. 3). It is not known if this receptor also binds the AHLs produced by YenI. In the genome of *P. luminescens*, two genes encoding putative AHL-LuxR-like receptors, *plu0320 *and *plu4562*, but no *luxI *genes are present (Fig. 3). This suggests that *P. luminescens *does not produce its own AHL signalling molecule, but might be able to sense those produced by other bacteria and therefore to detect mixed microbial communities as demonstrated for *Salmonella enterica *and *Escherichia coli *[35-37]. A similar function in *Y. enterocolitica *might be provided by YE1026. It is interesting to note that *Sodalis glossinidius *strain *morsitans*, an endosymbiont of the tse tse fly *Glossina morsitans morsitans *[38], also has two pontential AHL-LuxR receptors, SG1740 and SG0285, but no *luxI *homologue (Fig. 3). Instead of producing AHLs to regulate quorum dependent genes, a common strategy of insect-colonizing bacteria might be the detection of AHLs as a signal for the presence of other bacteria such as those colonizing the insect intestinal tract or living in soil.

**Table 1:** Low-temperature induced genes and proteins and their putative function during the bacterial lifestage in insects. The differential expression was observed in (1, 2, 8, 9, 10, 12) *Y. enterocolitica *[7, 78, 87, 94, 147, 148], (3, 4, 7) *Y. pestis *[149-151], (5) *Y. ruckeri *[152], (11) *Y. pseudotuberculosis *[153] and (6) *P. luminescens *[154].

**Class**	**Name**	**Function**	***Y. enterocolitica***	***P. luminescens***	**Distribution among bacterial genera**	**Possible role in insect gut/hemolymph**
**Substrate transport**	*gltP*/*dctA *(1)	glutamate-aspartate symport/transport of C4- dicarboxylates across the membrane	0310 (*gltP*)/4067 (*dctA*)	*dctA *(Plu3205)	ubiquitous	uptake of peptides following protease activity
	- (1)	permease	YE3697	Plu4591	ubiquitous	unknown
	*uhpABC *(1)	hexose phosphate transport	YE4089-4087	Plu0815-0813	ubiquitous	exploitation of carbon sources
	*mgtC *(1)	Mg^2+ ^transport ATPase protein	YE2586	Plu1843	ubiquitous	virulence factor in Salmonella
	*hemHFRS *(3, 4)	hemin storage	YE2481-2484	no homologue	ubiquitous	storage of excess hemin
	*irp1/irp2 *(7)	yersiniabactin biosynthesis	YE2617/YE2618	Plu2320/Plu2321	ubiquitous	iron acquisition
	*fepG *(5)	iron-siderophore transport	YE3620	Plu4625	ubiquitous	iron acquisition
**Membrane proteins**	- (1)	unknown	YE1324	no homologue	ubiquitous	unknown
	*ompN *homologue (1)	pore formation	YE2463	Plu1751	ubiquitous	osmolarity
	*crcB *(1)	unknown	YE0964	Plu1290	ubiquitous	unknown
	- (1)	unknown	YE2063	no homologue	*Yersinia*, *Burkholderia*, *Pseudomonas*	unknown
	- (1)	hypothetical membrane protein	YE2063	no homologue	*Yersinia*, *Burkholderia*, *Pseudomonas*,	unknown
	*rfb *(9)	synthesis of LPS O antigen	YE3072-3087	Plu4817-4819, Plu4824, Plu4831	ubiquitous	blocking the access of bile salts and complement to the outer membrane
	- (1)	putative lipoprotein	YE2793	no homologue	species-specific	unknown
**Substrate utilization**	urease (1, 3, 10)	urea amidohydolase	YE0951-0958	Plu2171-2177; transporter missing	ubiquitous	pH adaptation, ammoniak degradation
	*hutH *(1)	histidine ammonia-lyase	YE3021/YE4094	Plu3192	ubiquitous	histidine utilization
	*prtA *(6)	alkaline metalloprotease	YE4052	Plu0655	*Yersinia*, *Serratia*, *Pseudomonas*, *Erwinia*	bioconversion
	*glgB *(1)	1,4-a glucan branching enzyme	YE4013	no homologue	ubiquitous	storage of surplus primary carbohydrates
**Regulation**	*arcAB *(1)	TCS controlling the response to respiratory conditions	YE0595	Plu0562	ubiquitous	(virulence) regulation during anaerobic growth
	- (1)	putative transcription regulatory protein	YE1436	Plu2862	*Yersinia*	unknown
	- (1)	EAL domain; hypothetical	YE4063	no homologue	*Yersinia*, *Shigella*, *Escherichia*	regulation of virulence via c-di-GMP
	- (1)	EAL domain; hypothetical	YE1324	no homologue	*Yersinia*, *Shigella*, *Escherichia*, *Vibrio*	regulation of virulence via c-di-GMP
	*yenI/yenR (yspI/yspR) *(3)	N-acylhomoserine lactone synthase YenI/transcriptional regulator YenR	YE1600/YE1599	no homologue	*Sodalis*, *Serratia*, *Erwinia*, *Aeromonas*, *Pectobacterium*, *Pseudomonas*, *Agrobacterium*,	quorum sensing contributing to the regulation of virulence gene expression
**Virulence factors**	*srfA *(1)	putative virulence factor, *ssrAB *activated in *S. typhimurium*	YE2057	no homologue	*Yersinia*, *Salmonella*, *Pseudomonas*, *Enterobacter*	unknown
	*fhaC *(1)	hemolysin secretion	YE0480	no homologue	*Yersinia*, *Burkholderia*, *Pseudomonas*, *Bordetella*, *Haemophilus*	cytolytic effect on immunocytes and hemolytic effect on blood cells
	*yst *(2)	heat-stable enterotoxin	not annotated	no homologue	*Yersinia*	release of nutrients from gut cells
	*tcdA, tcdB *and *tccC*-like elements (1)	insecticidal toxin complex	*tc*-PAI^*Ye*^	numerous loci	*Yersinia*, *Xenorhabdus*, *Serratia*	cytotoxic activity against insect tissue
	*phlA/B *(5)	hemolysin secretion	YE2407/YE2408	Plu0316/Plu0317	ubiquitous	cytolytic effect on immunocytes and hemolytic effect on blood cells
	*invE *(11)	adhesion/invasion	YE3547	no homologue	ubiquitous	colonization of insect gut
	*ysa *(12)	T3SS	see Fig. 5	see Fig. 5	*Yersinia*, *Burkholderia*, *Erwinia*, *Xanthomonas*, *Salmonella*	colonization of insect gut
	*fleABC etc*.(1)	flagellar genes	Flag-1 genes	Flag-1 genes	ubiquitous	motility
	*yplA *(8)	Phospholipase	YE1005	Plu3370	*Yersinia*, *Serratia*, *Xanthomonas*	survival within the insect host

##### Regulation by AI-2

Beside AHL, other putative quorum sensing signalling molecules have been identified. One of them is autoinductor 2 (AI-2), furanosyl borate diester, which is synthesized by the *luxS *product [39,40] of which homologues are present in *P. luminescens *and *Y. enterocolitica *(*plu1253, ye0839*). It has been shown that the *luxS *pathway negatively controls the expression of genes for carbapenem antibiotic biosynthesis in *P. luminescens *[41]. Overall, more than 300 AI-2 regulated genes involved in regulation, metabolic activity, stress response and pathogenicity are known in *P. luminescens *[42]. For example, the expression of *tcdA1 *and *tccC1 *encoding subunits of the insecticidal toxin complexes and the production of *mcf2 *encoding the "Makes caterpillar floppy" toxin was identified to be *luxS *dependent. The Δ*luxS *mutant also showed decreased expression of virulence factors such as the cytotoxic protein CcdB, the hemolysin secretion protein HlyD (Plu0635), and the toxin ABC transporter subunits RtxD/RtxB. Homologues of these proteins are present in *Y. enterocolitica *(see chapter 2), suggesting their possible regulation by AI-2 also in this bacterium. Furthermore, the *P. luminescens luxS*-negative strain exhibited decreased biofilm formation, increased type IV/V pilus-dependent twitching motility, and attenuated virulence against insect larvae [42]. Taken together, a similar and AI-2 dependent mechanism for the regulation of insect colonization and insect pathogenicity might be used by both organisms. Whether they sense self-produced or external AI-2, or a combination of both, indicating a quorum sensing mechanism or a regulation similar to AHL as described above, remains to be elucidated.

##### Regulation by PAS_4/LuxR-like receptors

In *P. luminescens*, the amount of *luxR*-like genes is overrepresented with 39 copies in the genome. 35 of these potential LuxR-like receptors exhibit PAS_4 signal binding domains instead of an AHL-binding domain, and two have a signalling domain with a yet unidentified motif (Fig. 3). Most of the genes are located in two large gene clusters (*plu0918-0925 *and *plu2001-2019*). Interestingly, eleven of those LuxR-like receptors are present in *Y. enterocolitica *of which five are located in a cluster (*ye0035-0039*). Nine of these have a so-called PAS_4 signal binding domains of yet unknown function. It is interesting to note that there is only one bacterium else, the insect colonizing *S. glossinidius*, whose genome also carries a series of unclustered genes coding for PAS_4/LuxR-like receptors, indicating that this kind of receptors plays a role during insect infection. PAS-domains have been suspected to act as insect juvenile hormone (JH) receptors in the fruit fly *Drosophila melanogaster *[43,44]. The methoprene-tolerant gene (*met*, also called Resistance to Juvenile Hormone Rst1JH) of *D. melanogaster *encodes a helix-loop-helix transcriptional regulator combined with a PAS_3 domain [45]. Met has been shown to bind JH at physiological concentrations and is therefore suspected to act as a JH receptor [46,47]. Therefore, the potential PAS_4/LuxR-like receptors of *P. luminescens*, *Y. enterocolitica*, and *S. glossinidius *might sense JH or other eukaryotic hormones of the insect to adapt their gene expression to the insect host. The high number of 35 highly homologous receptor proteins in *P. luminescens *might be the reason for the wide insect host spectrum this pathogen is capable to infect. Although *Y. enterocolitica *protein extracts confer toxicity against *M. sexta *larvae [7], its host spectrum still remains to be defined. The difference in the number of the uncommon LuxR-like receptors (35 in *P. luminescens*, nine in *Y. enterocolitica*) gives rise to speculations that the insect host spectrum is constricted for *Y. enterocolitica *compared with *P. luminescens*. This hypothesis is underlined by the fact that not more than five PAS_4/LuxR-like receptors are present in *S. glossinidius *for which only one insect host has been reported.

##### Regulation by uncommon LuxR-like receptors

LuxR-like receptors in *Y. enterocolitica *with a yet unidentified signalling binding-site are YE2705 and YE3014, both of which are also present in *Y. pestis *(YPO2955 and YPO2593) and in *S. glossinidius *(SGP1_007, SG1174, SG1480, and SG1698), but not in *P. luminescens *(Fig. 3). It might be possible that signalling molecules of mammals and hormones of adult insects are sensed via these receptors by *Y. enterocolitica *and *S. glossinidius*, respectively, hosts which *P. luminescens *does not specifically interact with during its life cycle. In *P. luminescens*, two LuxR-like receptors with a yet unidentified signalling binding site are present, Plu4274 and Plu1817, the latter of which is shared by *Y. enterocolitica *(YE1621), but not by *S. glossinidius*.

#### Universal stress proteins

Universal stress proteins (Usp) are small soluble proteins found in bacteria, archaea and plants. The production of these proteins is induced upon global stress conditions such as nutrient starvation, heat stress, osmotic stress, oxidative stress, or the presence of toxic compounds. The protein family is divided into the UspA subfamily and the UspFG subfamily. The functional mechanism of these Usp proteins is not known [48]. Because *P. luminescens *and *Y. enterocolitica *are exposed to those stresses upon infecting and colonizing the insect host, we compared their set of Usp proteins (Table 2). Both genomes share an UspA-like (Plu0121 and YE4050) and an UspE-like (Plu2178 and YE2076) homologue. In *E. coli*, the sequence motif of Usp proteins is not highly conserved: UspA and UspC show a sequence identity of 37% and a homology of 57%, for example. In contrast, the UspA and the UspE homologues of *P. luminescens *and *Y. enterocolitica *are nearly similar, indicating that an identical stress response is regulated by these proteins. Homologues of these proteins are also present in *P. aeruginosa*, namely PA4352 and PA3309, a tandem-type Usp protein and a UspA-like protein, respectively. They are essential for survival under anaerobic growth and therefore biofilm formation, a situation cells are exposed to when colonizing the cystic fibrosis lung in hosts [49,50]. The Usp homologues of *P. luminescens *and *Y. enterocolitica *might also be important during infection of the insect host.

**Table 2:** Universal Stress Proteins (Usp) in *P. luminescens *and *Y. enterocolitica*

**Name**	***Y. enterocolitica***	***P. luminescens***	**Coherence with insect association**
UspA	YE4050	Plu0121	infection, colonization, anaerobiosis? switch to pathogenicity
UspE	YE2076	Plu2178	infection, colonization, anaerobiosis?
UspC	YE2583	no homologue	?
UspG	no homologue	Plu2030, Plu2032	?

In *P. luminescens*, expression of UspA has been shown to be under control of the AstS/AstR TCS, which is important for the correct timing of phase variant switching [28]. It is discussed that the AstS/AstR-system prevents or delays phenotypic variation by protecting the cell from stress [18]. Because *Y. enterocolitica *produces the corresponding TCS BvgS/BvgR, but is not known to switch to another phenotypic variant, the possible role of UspA in global regulation still remains to be elucidated. Phenotypic variation and thus the switch between mutualism and pathogenicity in *P. luminescens *is proposed to be regulated by a Ner-like and a HexA-like regulator that repress primary variant specific genes in the stage of the secondary variant [17]. Therefore, UspA might have a global importance in *P. luminescens *notifying stress and transmitting signals for HexA [18]. In *Y. enterocolitica*, the transcriptional repressor RovM (YE1343) is similar to HexA of *P. luminescens *(61% identity and 75% homology), and has only recently been shown to control cell invasion, virulence and motility in *Y. pseudotuberculosis*, *Y. pestis *and *Y. enterocolitica *[51-53]. This fact suggests a similar UspA-dependent regulatory mechanism used by the two bacteria compared here.

*P. luminescens*, but not *Y. enterocolitica*, produces two members of the UspFG family, the UspG homologues Plu2030 and Plu2032 (Tab. 2), indicating a global stress response induced by those Usp proteins that is different in both organisms. It is known that UspG of *E. coli *interacts with the chaperonin GroEL [54], which promotes the correct folding of many cytosolic proteins [55]. A GroEL homologue is present in *P. luminescens *(Plu4134) which the *P. luminescens *UspG homologues might interact with. In contrast to *P. luminescens*, *Y. enterocolitica *encodes another member of the UspA subfamily, the UspC homologue YE2583 (Tab. 2), which is not present in *P. luminescens*. Therefore, an UspC mediated stress response is not assumed to play a major role in insect pathogenicity.

Summarizing, the set of the shared and different Usp proteins reveals a partially similar and a partially different (fine)-regulation of the global stress response modules in *P. luminescens *and *Y. enterocolitica*. This pattern corresponds to the overlapping life cycles of both pathogens (Fig. 1). The UspA and the UspE homologues are predicted here to be relevant for insect infection, whereas UspC is assumed be more important for *Y. enterocolitica *in other environments/hosts. The two UspG homologues might constitute a set of Usp proteins that play a specific role in *P. luminescens *infection or in symbiosis with the nematode host.

#### Regulation via c-di-GMP as a second messenger

Cyclic diguanylate (c-di-GMP) is a bacterial second messenger that activates biofilm formation while inhibiting motility, thus regulating the switch between a planktonic and a sessile lifestyle. In addition to phenotypes that affect virulence properties indirectly, c-di-GMP can also directly regulate virulence factors [56,57]. Proteins containing a so-called GGDEF domain are responsible for the synthesis of c-di-GMP, and those with a so-called EAL domain for its degradation. The expression and activity of those GGDEF and EAL domain containing proteins is regulated by factors with a PilZ domain that binds c-di-GMP. The PilZ domain is found as a stand-alone domain or in combination with GGDEF, EAL and other domains, thus assumed to function also as an allosteric domain to control other regulatory enzymes [58,59]. In *Y. enterocolitica*, we identified 22 putative proteins containing GGDEF and EAL domains. Eleven of these proteins solely contain a GGDEF-domain and six solely an EAL-domain, and both domains are found in tandem in five proteins. The protein AdrA (YE3010, GGDEF domain) is annotated as a putative diguanylate cyclase, YE2278 (GGDEF+EAL) as a putative phosphodiesterase, YE3818 (GGDEF) as a putative regulator, and YE3806 (GGDEF+EAL) as a putative exported protein. All other GGDEF and EAL domain-containing proteins are of unknown function. Furthermore, two proteins with PilZ domain exist in *Y. enterocolitica*, namely YE3197 and BcsA (YE4074), a putative cellulose synthase. Cellulose synthesis in bacteria has been identified to be important for the protection from chemical or mechanical stress by forming a hydrophobic extracellular matrix [60]. The expression of two of those EAL-domain containing proteins, YE4063 and YE1324, is induced at low temperature (Tab. 1). These two factors might therefore be important for insect colonization instead for virulence against mammals. The presence of c-di-GMP mediated regulation in *Y. enterocolitica *is therefore suggested to play a central role in switching from biofilm formation to the human as well as to the insect environment. *P. luminescens *contains no protein with GGDEF, EAL or PilZ domain. This phenomenon is quite surprising, because with few exceptions such as *Helicobacter pylori*, nearly all pathogenic bacteria use c-di-GMP as a second messenger. It has been reported that *P. luminescens *forms biofilms *in vitro*, and that a *luxS*-deficient mutant unable to synthesize the quorum-sensing inducer AI-2 showed a decreased biofilm formation [42]. The lack of these protein domains in *P. luminescens *reveals that c-di-GMP signalling plays a major role in pathogenic bacteria when colonizing a mammalian host, and a minor for invertebrate colonization of entomopathogenic or entomoinfecting bacteria.

### Virulence factors

**Figure 4:**
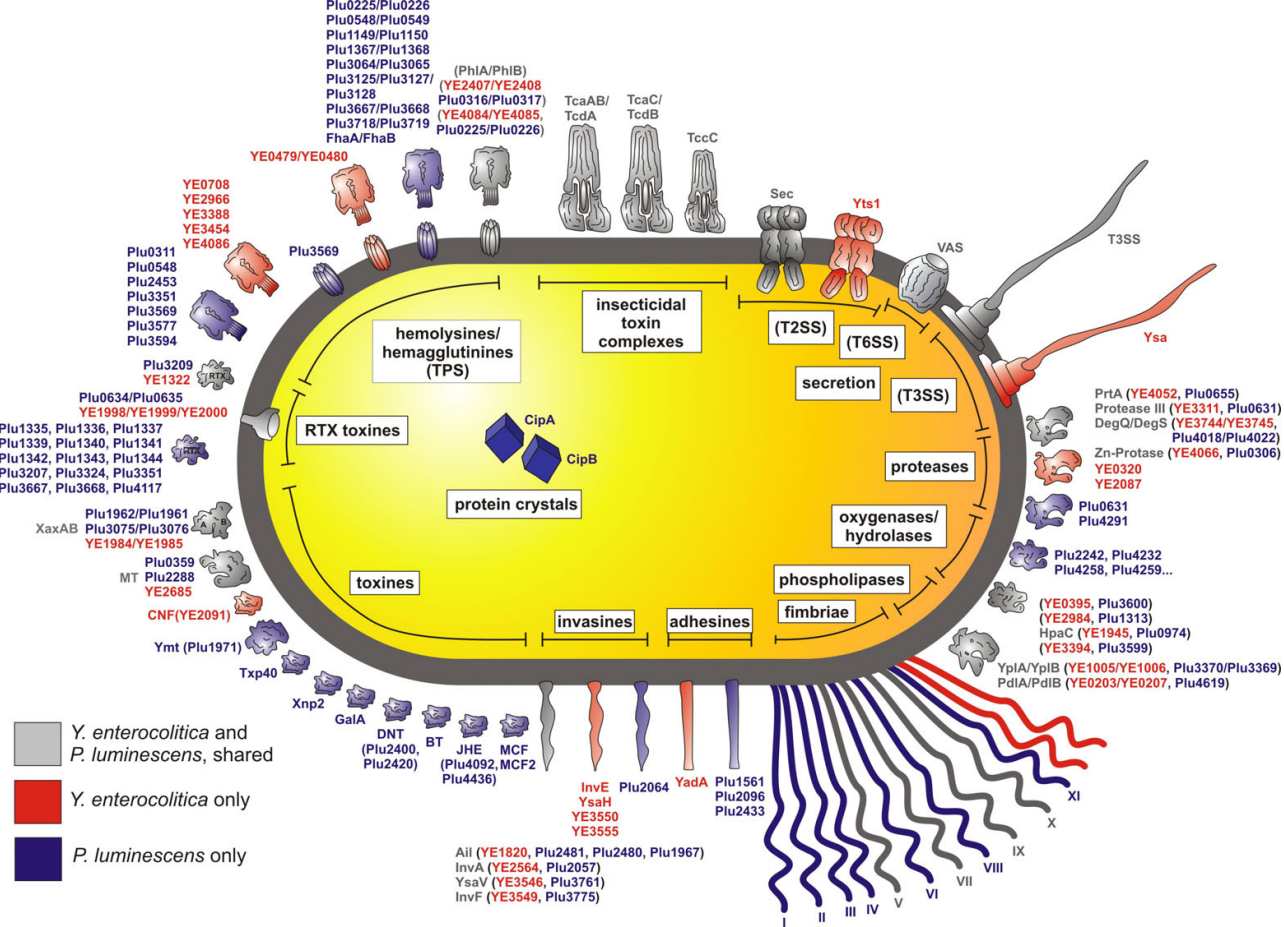
**Virulence factors in *P. luminescens *and *Y. enterocolitica***. The different toxins shared by the two organisms are presented in grey colour, toxins only present in *P. luminescens *or in *Y. enterocolitica *are depicted in blue or in red, respectively. DNT: Dermonecrotic Toxin, BT: *Bacillus thuringiensis *like toxin crystal, JHE: Juvenile Hormone Esterase, MCF: "Makes Caterpillars Floppy", MT: Macrophage Toxin, CNF: Cytonecrotic Factor, RTX: "Repeats in toxin", Ymt: *Y. pestis *murine toxin. The toxins are grouped in functional classes, and the respective homologues in *P. luminescens *and *Y. enterocolitica *are indicated. See text for further details.

So-called offensive virulence factors actively contribute to a successful infection by colonization of and toxicity towards the host organism. We compared both genomes with respect to genes encoding toxins, adhesins or invasines that are common to both pathogens. All virulence factors described in the following are summarized in Fig. 4.

#### Toxins

##### Insecticidal toxins

The insecticidal toxin complex (Tc) proteins were first purified from *P. luminescens *[61]. Tc homologues have also been described to be present in *Yersinia *spp. and in other insect-associated bacteria such as *Serratia entomophila *and *Xenorhabdus nematophilus *[62,63]. The respective genes encoding four high molecular weight toxin complexes are termed *tca*, *tcb*, *tcc *and *tcd*. Further experiments supported the hypothesis that TccC-like proteins might act as universal activators of, or chaperons for, different toxin proteins, while Tca-like and Tcd-like proteins contribute predominantly to the oral toxicity of bacterial supernatants [17]. It is speculated that the Tc toxins are active against different tissues within individual hosts, namely Tcb against hemocytes and Tcd and Tca against cells of the insect gut. In *Y. enterocolitica*, the insecticidal toxin genes are located on a distinct genomic island termed *tc*-PAI^*Ye *^of 21 kb, and are low-temperature induced [7]. Similar islands in which regulatory genes are followed by three *tca *genes, phage-related genes and one or two *tccC *genes, are present in the genomes of *Y. pseudotuberculosis *IP32953 and *Y. pestis *KIM. In *P. luminescens*, the insecticidal genes are organized in the *tcd *island harbouring nine *tcd*- and *tcc*-like genes and several non *tc*-like genes, while further nine *tcc*-like genes are scattered over the chromosome [24]. The reason for the over-represence of *tc*-like genes in the *P. luminescens *genome might reflect the different strategies followed by both bacteria within insects, namely the rapid killing for exploiting the victim as a food source in case of *P. luminescens*, and infection of and persistance within the invertebrate host as possibly preferred by *Y. enterocolitica*.

##### Hemolysins or hemagglutinin-related proteins

These extracellular toxins target red blood cells to provide access to iron, but often show activity against immune cells, thus contributing to the bacterial response to the immune system of hosts, including phagocytosis by insect blood cells [64]. Hemolysins or surface-associated adhesins, together with their transporters, are sometimes organized as two-partner secretion (TPS) systems, a specialized mechanism for the delivery of large exoproteins [65]. TPS systems have been characterized mainly in pathogenic bacteria, but are also present in other microorganisms. *P. luminescens *and *Y. enterocolitica *TPS systems include the calcium-independent hemolysin PhlA that is transported through the outer membrane and activated by PhlB. Remarkably, their expression is induced by low iron concentration as encountered in the insect host, and *phlA*/*phlB *are up-regulated at 18°C compared to 28°C in *Y. ruckeri *[66]. Eight other TPS systems are present in *P. luminescens*, namely Plu0225/Plu0226, Plu0548/Plu0549, Plu1149/Plu1150, Plu1367/Plu1368, Plu3064/Plu3065, Plu3125-3127/3128, Plu3667/Plu3668, and Plu3718/Plu3719, and further three genes for which the partner locus has not been identified (Fig. 4). In the genome of *Y. enterocolitica*, only three complete TPS systems are present, namely YE0479/YE0480, YE2407/YE2408, (YE4084)YE4085/YE4086, and YE3454 which lacks the activator partner. Except YE0479/YE0480, all have counterparts in the *P. luminescens *genome. Recently, we have shown that a luciferase reporter insertion into YE0480 is induced at low temperature [67], indicating that this TPS system might contribute to insect pathogenicity and possibly to the host-specificity of *Y. enterocolitica*. The genomes of both pathogens also carry three and five, respectively, further hemolysin/hemagglutinin-related proteins which are absent in the other pathogen (Fig. 4). FhaC which belongs to a family of hemolysin activator proteins related to ShlA from *Serratia marcescens *is present in both pathogens and also induced at low temperature [67]. The genome sequence of *P. luminescens *exhibits more toxin genes than found in any other bacterial genome sequenced yet, including the genome of *Y. enterocolitica*. Hemolysin-related factors and their transporters discussed above are an example for this redundancy. However, the majority of these *P. luminescens *toxins exhibit highly significant similarities to those of *Y. enterocolitica*, suggesting common progenitors of hemolysins. It is therefore tempting to speculate that hemolytic activities of bacteria had been evolved during the association with insects and then adapted to mammalian hosts. Although it can not be excluded that the hemolysins of *Y. enterocolitica *act on the immune systems of both the insect and the mammalian host, the genetic overlap of this group of virulence factor between both pathogens, and the low-temperature expression of YE0479/YE0480 and *fhaC*, indicates the presence of insect-specific hemolysins in the genome of *Y. enterocolitica*.

##### Repeats-in-toxin (RTX) and other toxins

RTX proteins constitute another family of toxins that may contribute to the insecticidal activity of the two pathogens. A putative RTX-family toxin transporter is common to both pathogens (YE1998-2000, Plu0634/Plu0635). The *P. luminescens *genome comprises a gene cluster encoding RTX proteins, namely *plu1330-1369*. Further RTX toxins are encoded by *plu3217*, *plu3324 *(both RTX A-family), *plu4117 *(own family), and *plu3668 *(RTX cytotoxin), none of which is present in *Y. enterocolitica*. This pathogen produces only one RTX protein (YE1322) for which a truncated homologue is found in *P. luminescens *(Plu3209).

Other examples of toxins common for both bacterial species compared here are homologues of XaxAB, an apoptotic AB toxin of *X. nematophila *[68], and proteins encoded by the macrophage toxin (*mt*)-like genes Plu2288 and Plu0359 with high similarity to YE2685. *cnf *encoding the cytonecrosis factor-like toxin is present in *Y. enterocolitica *(YE2091) and *P. luminescens *ssp. *akhurstii *strain W14, but not in *P. luminescens *ssp. *laumondii *strain TT01 (Fig. 4). *P. luminescens *produces a series of proteins similar to toxins that have been identified in other bacteria, but are absent in *Y. enterocolitica*. Examples identified are Txp40, a 40 kD insecticidal toxin [69], the nematicidal toxin (Xnp2) first described in *X. bovienii *(accession number AJ296651.1), *galA *(*plu0840*) similar to the enterotoxin Ast of *Aeromonas hydrophila *which is involved in carbohydrate transport and metabolism [70], and two dermonecrotizing toxin-(*dnt*-) like factors (*plu2400 *and *plu2420*). In addition, neither the crystal proteins encoded by *cipA *and *cipB *in *P. luminescens *nor a Bt-like toxin (*plu1537*) could be found in *Y. enterocolitica*. A cytonecrosis factor (CNF)-like protein, Pnf, was identified in *P. luminescens *ssp. *akhurstii *strain W14, but not in *P. luminescens *ssp. *laumondii *strain TT01. In *P. luminescens*, the two paralogs *plu4092 *and *plu4436 *encode juvenile hormone esterases (JHE) for which insect toxicity has already been demonstrated [24]. Additionally, neither the locus *mcf *that confers insecticidal activity of *P. luminescens *towards *M. sexta *[71] by inducing apoptosis [72], nor the homologous gene locus *mcf2 *(*plu3128*) [73] are present in the genome of *Y. enterocolitica*. Most of these toxins probably contribute to the higher insect toxicity of *P. luminescens *against the tobacco hornworm in comparison with *Y. enterocolitica*. No homologues of the *Y. pestis *gene coding for enhancin (YPO0339) could be found for which a role in flea colonization was predicted [74].

We also identified several virulence genes and operons that are present in *Y. enterocolitica*, but not in *P. luminescens*, suggesting that they have been acquired by horizontal gene transfer from other bacteria and do not play a role in bacteria-insect association. Examples are SopB, a host cell invasion factor translocated via the type-III secretion system that is present in the emerging human pathogen *P. asymbiotica*, but not in the insect pathogen *P. luminescens *[14], a putative effector protein (YE2447) with proteolytic activity, and a homologue of SrfA which is negatively regulated by PhoP in *S. typhimurium *[75]. The SrfA homologue has been demonstrated to be up-regulated by environmental temperature [67]. Other virulence factors absent in *P. luminescens *are the *opg *cluster (YE1604-1606) and ProP (YE3594), both involved in osmoprotection [76], cellulose biosynthesis (YE4072-4078) associated with protection from chemical and mechanical stress [60], the methionine-salvage pathway (YE3228-3235) also involved in AHL production [23], the putative ADP-ribosyltransferase toxin encoded by *ytxAB *(*ye2124*/*ye2123*) [77], and the *Yersinia *heat-stable toxin Yst [78] which is stronger expressed at 28°C than at 37°C (Table 1).

**Figure 5:**
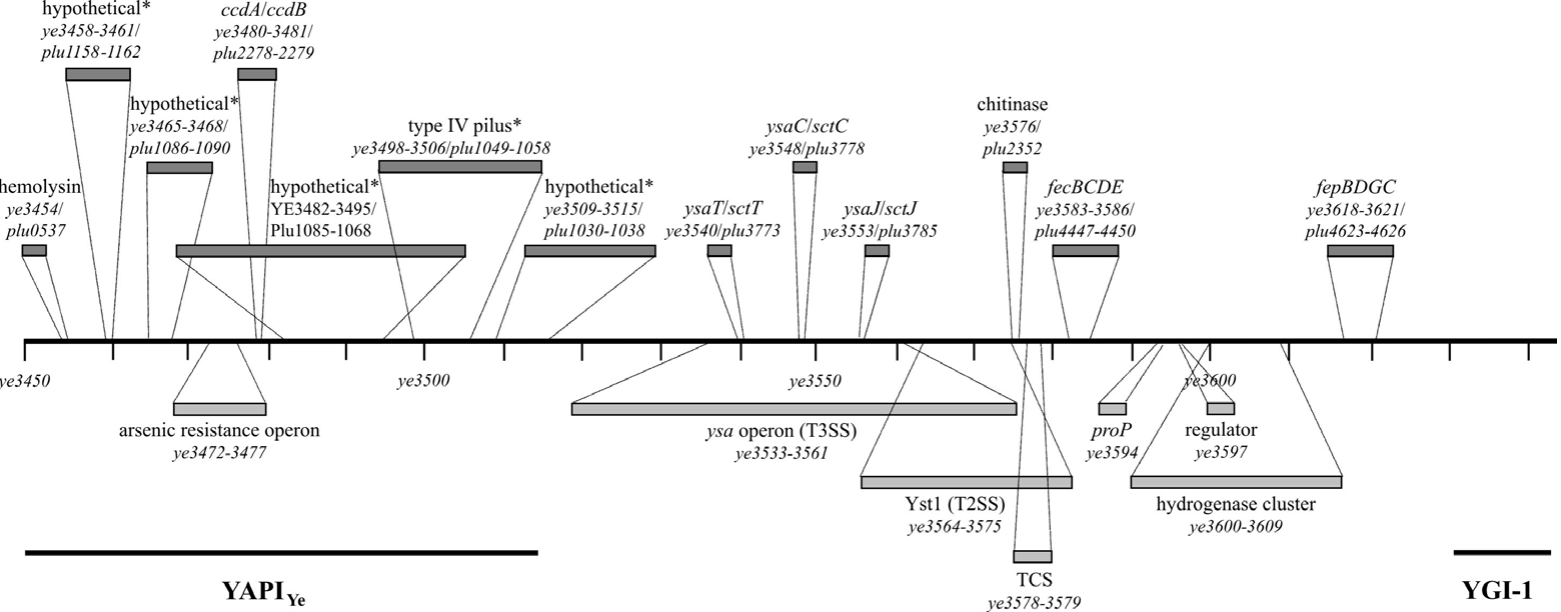
**Plasticity zone of *Y. enterocolitica *(YE3450-3644) compared to the *P. luminescens *genome**. YAPI_Ye_, a genomic region highly similar to the adhesion pathogenicity island of *Y. pseudotuberculosis *and absent in *Y. pestis*. Sites marked with an asterisk are localized on genome island Plu0958-1166. In *Y. enterocolitica *biovars 2–5, a second flagellar gene cluster (Flag-2) is inserted next to *ye3610 *[120]. Genes, operons or proteins shared by *Y. enterocolitica *and *P. luminescens *are depicted in dark grey colour above the chromosomal scale, and genes without homologue in *P. luminescens *are shown in light grey below the line. See section "Evolution of pathogenicity" for further details.

Summarizing, the large variety of diverse toxins present in *P. luminescens*, but absent in *Y. enterocolitica*, might contribute to the higher toxicity towards insects of *P. luminescens *in comparison to *Y. enterocolitica*. Toxins only present in *Y. enterocolitica *are assumed to play a major role in its pathogenicity towards mammalians, and some of them might have been acquired by horizontal gene transfer. Examples of those factors are shown in Fig. 5.

#### Adhesins and invasins

Colonization and penetration of epithelial cells, and interaction with immune cells, are key steps during the host infection by pathogens. Many of the pathogen-receptor molecules such as Toll-like receptors or integrins are conserved between invertebrates and mammalians [79]. We therefore investigated if *P. luminescens *and *Y. enterocolitica *that interact with the midgut of diverse hosts use the same adhesion and invasive factors. The most prominent protein of *Y. enterocolitica *involved in attachment to and invasion of mammalian cells is Ail (YE1820) that is homologue to three *P. luminescens *proteins encoded by *plu2481*, *plu2480*, and *plu1967*, and InvA (YE2564) with high similarity to Plu2057. Further invasin genes of *Y. enterocolitica *with counterparts in *P. luminescens *are *ysaV *(*ye3546*/*plu3761*) and *invF *(*ye3549*/*plu3775*). *Y. enterocolitica *genes not present in *P. luminescens *are *ye1873 *encoding the adhesin YadA which is maximally expressed at 37°C, and the invasin genes *invE*, *ysaH*, *ye3550*, and *ye3555*. The function of the latter two in cell recognition is predicted, but has not yet been demonstrated experimentally. In contrast, *P. luminescens *produces several factors involved in host cell interaction without homologues in *Y. enterocolitica*, namely Plu2096 which is similar to lectin PA-I, Plu1561 with strong homology to a Ca^2+ ^dependent adhesion molecule, the adhesin Plu2433 similar to a virulence factor of the Gram-negative plant pathogen *Erwinia carotovora*, EvF, which is involved in colonisation of the *D. melanogaster *gut epithelium [80], and the putative invasin Plu2064. In *P. luminescens*, eleven fimbrial gene cluster have been identified, four of which (V, VII, IX and X) are also present in *Y. enterocolitica*. Unique for the human pathogen in comparison to *P. luminescens *are the two fimbrial gene cluster *ye2664*-*2668 *and *ye2692*-*2700*, both of yet hypothetical function. Thus, invasin and adhesin homologues similar in the two pathogens might contribute to the infection of insect or mammalian hosts, but candidates for insect- and mammalian-specific colonization factors have also been revealed by the genome comparison performed here.

#### Defensive mechanisms

##### Antimicrobials

The production of antibiotics is mainly restricted to *P. luminescens*, whereas factors combating antimicrobial host substances play an important role during the infection process of both pathogens compared here. In the genome of *P. luminescens *ssp. *laumondii *strain TT01, many loci involved in the defense of the insect cadaver against different microbial competitors are present, including nearly 50 genes encoding proteins such as polyketide and peptide synthases putatively involved in antibiotic synthesis and efflux. Interestingly, none of these genes showed significant similarities to sequences of the *Y. enterocolitica *genome. Phage-derived bacteriocins in entomopathogenic bacteria are also presumed to eliminate competing bacteria. More than twenty colicin/pyocin-like factors and putative immunity proteins are unique to *P. luminescens *in comparison to *Y. enterocolitica*. Remarkable exceptions are the toxin/antitoxin system *ccdA*/*ccdB*, the *tolQRAB*/*pal *operon involved in group A colicin translocation, and a colicin production and secretion system (Plu3168/Plu3869; YE0791/YE1314). Recently, it was reported that PrtS (Plu1382) secreted by *P. luminescens*, a metalloprotease without counterpart in *Y. enterocolitica*, specifically induces melanization of the hemolymph, probably to circumvent the innate immune response of the insect [81].

##### Oxygenases and hydrolases

*P. luminescens *produces proteins similar to monooxygenases, dioxygenases and hydroxylases that have been suggested to play a role in rapid elimination of insect polyphenols or in the detoxification of reactive oxygen species generated by the invaded host [24]. Examples are the product of *plu4258*, adjacent to a gene encoding glutathione transferase (*plu4259*), a putative steroid monooxygenase (Plu4232), and a glycine oxidase (Plu2242), all of which have no counterparts in *Y. enterocolitica*. Factors present in both pathogens are two monooxigenases encoded by *ye1945*/*hpaC *(*plu0974*) and *ye3394*/*plu3599*, and two hydroxylases encoded by *ubiH *(*ye3395*/*plu3600*) and *ubiF *(*ye2984*/*plu1313*). It is therefore possible that the *Y. enterocolitica *homologues of these enzymes are involved in persistence within the insect, a mechanism which is also used by *P. luminescens*.

#### Secretion and exoenzymes

In *Y. enterocolitica*, two type-III secretion systems (T3SS) essential for virulence in the mammalian host are encoded on pYV and by the *ysa *operon (YE3533-3561) [23,82]. The *P. luminescens *genome encodes one T3SS which is highly similar to the plasmid-encoded T3SS of *Y. enterocolitica *and probably involved in the secretion of virulence proteins or in immunomodulation of the insect response to an infection. Interestingly, the T3SS of *Y. pestis *has recently been demonstrated to translocate insecticidal toxins, providing evidence that they support the transmission of the plague agent by insects [83]. Furthermore, the flagellar export apparatus of *Y. pseudotuberculosis *functions as a secretion system for the virulence-associated phospholipase YplA [84]. The typical effector proteins of *Y. enterocolitica *are also present in *P. luminescens*. The *P. luminescens *Lop effector proteins are homologs of the Yop effector proteins of *Y. enterocolitica *[85]. The LopT effector protein of *P. luminescens *can be injected by *Y. enterocolitica *into mammal cells [86], underlining the idea that both T3SS act similarly. Furthermore, we found homologues of the *Y. enterocolitica *low-calcium-response genes (*lcrH*, *lcrV*, and *lrcD*) in *P. luminescens *(*plu3757*, *plu3758*, *sctV*) which further supports this hypothesis. The fact that *Y. enterocolitica *a second T3SS (Ysa) is not shared by *P. luminescens *confirms its solely role in human pathogenicity [87].

Both *P. luminesens *and *Y. enterocolitica *share a Sec protein translocation system that belongs to the type-II secretion systems (T2SS). These are substrate-specific secretion machineries that share a similar architecture and secretion mechanism [88]. Proteins secreted by these systems are mainly virulence determinants such as exotoxins like the Cholera toxin of *Vibrio cholerae*, pili, and S-layer components (see [89] for review). Additionally to the Sec-system, *Y. enterocolitica *produces a T2SS named Yts1, which has been found to be important for virulence in mice [90]. Because there is no counterpart of Yts1 present in *P. luminescens*, one can speculate that the major parts of type-II dependent secreted proteins which are important for insect association of *Y. enterocolitica *are translocated via the Sec system.

Recently, a novel protein secretion mechanism translocating proteins without an N-terminal leader sequence has been described, termed type-VI secretion system, T6SS (see [91] for review). The genes encoding these kinds of secretion systems were named *vas *(virulence associated secretion), and homologues are widespread in Gram-negative bacteria. VAS-dependent secretion has been found to be important for virulence of *Vibrio cholerae *[92] as well as for *Pseudomonas aeruginosa *[93], and T6SS are assumed to play a major role in virulence in many Gram-negative bacteria [91]. *P. luminescens *as well as *Y. enterocolitica *harbour homologues of the *vas *genes, indicating that several proteins involved in virulence are secreted via this pathway.

Both pathogens secrete lipases and proteases that are assumed to contribute to immunosuppression, degradation of insect tissues or antibacterial peptides, and host bioconversion (Fig. 4). One of those exoenzymes is the phospholipase A (YplA) with an accessory protein (YplB) of *Y. enterocolitica *(YE1005/YE1006) which are also present in *P. luminescens *(Plu3370/Plu3369). YplA contributes to pathogenesis of *Y. enterocolitica *in a mouse model [94], suggesting a role in virulence against insects for the *P. luminescens *homologue. Remarkably, *yplA *is induced at low temperature (Table 1), and its expression is known to be regulated by the master regulator FlhDC [94], indicating that YplA plays a role in pathogenicity both against human and insect hosts. Two additional phospholipases are present in *Y. enterocolitica*, namely PdlA (YE0203) and PdlB (YE0207), the latter one a homologue of Plu4619. This overlap is another example for *Y. enterocolitica *enzymes probably involved rather in the association with invertebrates than in pathogenicity towards mammalians. Plu1971 of *P. luminescens *is a protein which contains two phospholipase D motifs. Furthermore, it shares homologies to the plasmid (pMT1)-encoded murine toxin (Ymt) of *Y. pestis*. It was suggested that *ymt *has been acquired by *Y. pestis *from *P. luminescens *or a close relative [24]. Ymt is essential for flea colonization by *Y. pestis *and is regulated by AHL both in *Y. pestis *[95] and *P. luminescens *(R. Heermann, unpublished data), indicating that Ymt is also required for insect colonization by *P. luminescens*. Due to its absence in *Y. enterocolitica*, Ymt is another example for the high diversity of genetic determinants that are used by closely related bacterial pathogens to interact with their insect hosts.

There are several other exoenzymes present either in *Y. enterocolitica *or in *P. luminescens*, which do not have a homologue counterpart in the other bacterium. Examples are the ten triacylglycerol lipases of *P. luminescens *or the three identified lipases of *Y. enterocolitica*. However, homologies are observed for six secreted proteases of both organisms. Among them is PrtA (Plu0655/YE4052), a zinc metalloprotease that is involved in the immunosuppressive activity of *X. nematophila *[96], and that has also been shown to be involved in insect gut colonization of *P. luminescens *[97]. Further examples for shared proteases are another Zn-dependent protease (Plu0306/YE4066), the protease III (Plu0631/YE3311), and DegQ/DegS (YE3744/YE3745/Plu4018/Plu4022). The high number of homologs in both organisms suggests an important and similar role of these exoproteases in the infection process. We also identified two proteases in each pathogen (Plu4291, Plu0631, YE0320, and YE2087) without a homologue in the other bacterium. We speculate that these *Y. enterocolitica *proteases could be involved in the infection process in mammals, whereas the *P. luminescens *proteases are rather used for nutrient bioconversion than for the infection process.

### Metabolism

While many specific virulence factors, which enable the microbes to overcome the various physical and biochemical barriers of the infected hosts, have been investigated in detail, little attention has been given to the metabolic requirements and substrate availability of bacteria *in vivo*. Both in insects and mammals, pathogens get access to host-specific nutrients, but also encounter substrate limitations such as low iron concentration. In this chapter, we focus on metabolic pathways of *P. luminescens *and *Y. enterocolitica *absent in *E. coli*, induced at low temperature, or already known to be virulence-associated.

#### Degradative pathways

*P. luminescens *and *Y. enterocolitica *share loci encoding several common degradation pathways that are absent in *E. coli *K-12, including the urease operon (*ureABCEFGD*), the genes involved in *myo*-inositol degradation, and the histidine degradation operon (*hutHUCGI*). These pathways might help the bacteria to gain access to sufficient amounts of substrates and thus to proliferate in the hemolymph of the insect larvae. We recently reported that the genes of the urease operon as well as a histidine ammonia lyase (*ye3021*/*plu1240*), which deaminates histidine to urocanic acid, are highly induced in *Y. enterocolitica *upon temperature decrease [67]. Beside arginine (5.17 μmol/g), lysine (12.23 μmol/g), serine (6.77 μmol/g) and proline (6.40 μmol/g), histidine (5.04 μmol/g) is the most abundant free amino acids in the *Hyalophora gloveri *fat body [98].

The synthesis of vitamin B12 that occurs only anaerobically is required for the degradation of 1,2-propanediol by the products of the *pdu *operon, as well as of ethanolamine by the *eutABC*-encoded enzymes. The cobalamine-dependent anaerobic growth of *Salmonella typhimurium *on both these substrates has been shown to be supported by the alternative electron acceptor tetrathionate whose respiration is facilitated by the tetrathionate reductase gene cluster *ttr *[99,100]. Beside *S. typhimurium*, all these genetic determinants were found only in few other bacteria, namely the human pathogens *Listeria monocytogenes*, and *Clostridium perfringens *[101]. *Y. enterocolitica *carries the genes encoding tetrathionate reductase (*ttrABC*) and the TCS TtrRS (YE1613-1617). The gene clusters for cobalamin synthesis and propanediol degradation are located on a 40-kb genomic island (*ye2707*-*2750*), but the *eutABC *operon is missing. Propanediol degradation by *Y. enterocolitica *might also be supported by YE4187 with a putative GlcG domain which is predicted to be involved in glycolate and propanediol utilization. The cobalamine synthesis genes and the *eutABC *operon, but not *ttrC, ttrR, ttrS *and the propanediol utilization gene cluster, are also present in the genome of *P. luminescens*, suggesting the degradation of phosphatidylethanolamine as additional energy source in the insect host [102].

Further metabolic genes common to both pathogens are *dctA *responsible for transport of C4- dicarboxylates across the membrane, the UhpABC regulatory system controlling the hexose phosphate transport by UhpT, and the three Mg^2+ ^transport systems CorA, MgtA and MgtB. The *uhpABC *operon as well as *mgtC *encoding the Mg^2+ ^transport ATPase subunit have been found to be induced at low temperature in *Y. enterocolitica *[67], indicating a relevance for these metabolic genes for *P. luminescens *and *Y. enterocolitica *during insect infection. Another gene, *gltP *encoding a glutamate-aspartate symporter, is also up-regulated at low temperature in *Y. enterocolitica*, but lacks a counterpart in *P. luminescens*. Furthermore, both insecticidal bacteria produce a chitin-binding-like protein (Plu2352, YE3576), but chitinase-like proteins (Plu2235, Plu2458 and Plu2461) are without homologues in *Y. enterocolitica*. This fact correlates once more with the separate lifestyle of both bacteria, e.g. association with the host and persistence for *Y. enterocolitica*, and association and bioconversion of the insect in case of *P. luminescens*.

#### Iron uptake

Bacteria use two different strategies to acquire sufficient amounts of iron, namely the expression and secretion of high-affinity iron-binding compounds called siderophores, and the production of receptors for iron carriers such as heme. Genes involved in the biosynthesis, transport and regulation of the siderophore yersiniabactin are clustered in the high pathogenicity island of *Y. enterocolitica *[103] and have counterparts in *P. luminescens *(*plu2316*-*2324*). Remarkably, yersiniabactin is absent in all *Y. enterocolitica *strains beside biovar 1B. Present in both bacterial organisms compared here are also genes encoding a hemine uptake system (*ye0323-0332*/*plu2631-2636*), the YfeABCD transporter system of chelated iron, the ferrous (Fe^2+^) iron transporter proteins FeoAB, the AfuABC/SfuABC ferric (Fe^3+^) transporter, the enterobactin and its transporter (FepBDCG), the FecABCDE ABC transporter system, and several putative hemin/siderophore/iron uptake proteins (YE1459-1461/Plu2850-2852), YE3190/Plu2853, and YE0555/Plu3738). The proteins encoded by the *P. luminescens fecIRABCDE *operon are similar to the components of the *E. coli *Fe^3+^-dicitrate transport system. Homologues are present in the genome of *Y. enterocolitica*, but scattered over the chromosome. In addition, *Y. enterocolitica *produces two heme-protein acquisition sytems (YE0123-126, YE2180-2182), a second SfuABC system, the ferrichrome binding and transport proteins (YE0730-0732), a putative siderophore (YE0704), and a hemin storage system (YE2481-2484). None of these iron acquisition systems is present in *P. luminescens *which in contrast produces the siderophore photobactin [104]. Furthermore, *P. luminescens *encodes two putative heme-binding hemopexin-like proteins, the photopexins PpxA (Plu4242) and PpxB (Plu4243), which are the first hemopexins found in bacteria. It is suggested that the photopexins may be used by *P. luminescens *to scavenge iron containing compounds from insects [105]. Interestingly, three gene loci involved in iron acquisition, namely the genes encoding the hemin storage system, the yersiniabactin and the enterobactin transporter FepG, have been demonstrated to be up-regulated upon temperature decrease in *Y. pestis *or *Y. ruckeri*, respectively (Table 1).

This large set of iron, hemin, heme and siderophore transporters underlines the importance of iron availability for the life cycles of *P. luminescens *and *Y. enterocolitica*. It also indicates that iron acquisition is a prerequisite for the infection process of pathogenic bacteria not only in mammalian, but also in invertebrate hosts, and underlines the suggestion that genetic determinants of invertebrate pathogens such as *P. luminescens *include the progenitors of virulence factors against vertebrates [79,106].

#### Tricarboxylate utilization

The TctE/TctD system is the only TCS of *P. luminescens *without homologue in *Y. enterocolitica *(see section "Two-component signal transduction", Fig. 2). It controls the expression of the *tctCBA *operon encoding the tricarboxylic acid transport system TctCBA [107]. The transporter is supposed to facilitate uptake of citrate, fluorocitrate, isocitrate and cis-acconitate for aerobic utilization [108,109]. The Na^+ ^dependent citrate symporter CitS of *S. enterica*, which the *Y. enterocolitica *protein YE2507 is homologous to, is induced by the CitA/CitB system for fermentation of citrate under anoxic conditions [110], indicating a general difference of citrate utilization in *P. luminescens *and *Y. enterocolitica*. While *Y. enterocolitica *explores citrate for anaerobic metabolism, it is most likely that the specific uptake of citrate and other tricarboxylic acids by TctCBA is used by *P. luminescens *upon entering the insect host where enough citrate is available. The specific up-regulation of the tricarboxylic acid cycle (TCA) enzymes within a host has also been described for other microorganisms. For example, *sucA *encoding a subunit of α-ketoglutarate synthase and *acnA *encoding the aconitase have been found to be induced in *V. cholerae *during host infection [111,112], and a complete TCA cycle is also required for *S. typhimurium *virulence [113]. We also observed induction of *sucA *in *P. luminescens *in the insect host *Galleria mellonella *(R. Heermann, unpublished data). This finding underlines the hypothesis that the citric cycle enzymes used under aerobic conditions are up-regulated as a specific adaptation of the metabolic activity in the nutrient rich insect host. To guarantee an optimal amount of tricarboxylic acids within the cell, TctE might specifically sense the presence of tricarboxylic acids and/or signals of the host. *Y. enterocolitica *and *Y. pestis*, in contrast exhibit upregulation of all TCA genes upon temperature shift from 26°C to 37°C [114,115]. Therefore, it is obvious that *Y. enterocolitica *and *P. luminescens *use different sensing and utilization strategies for tricarboxylates.

### Temperature-dependent genes

Temperature is a key environmental signal to enable bacterial adaptation to diverse hosts. In *Yersinia*, temperature-dependent gene expression has been described to be an important theme in bacterial mechanisms of pathogenesis towards humans [116]. However, the biological role of genes repressed at body temperature, but induced at environmental temperature, has been underinvestigated so far. By data mining, we identified 32 genes or gene loci of *Yersinia *spp. that exhibit stronger expression with temperature decrease (Table 1). 19 of them have a homologue in *P. luminescens*. Most genes belong to the groups of offensive virulence factors, regulators, and metabolic enzymes. The data have derived from expression profiling *in vitro *and cannot directly be translated to the *in vivo *situation. Moreover, several genes induced at lower temperature such as *inv*, *yst *or *yplA *affect the virulence properties of *Y. enterocolitica *in mice [87]. However, low temperature-dependent expression of the genes in Table 1 suggests that they also play a role during the insect stage of *Y. enterocolitica*, or that they have evolved from bacteria-insect interaction and then adapted to pathogenicity towards mammals. Some of these low temperature-induced genes are restricted to a narrow spectrum of bacterial genera such as *Burkholderia*, *Pseudomonas*, *Serratia*, or *Erwinia*, all of which are known to be associated with soil, plants or insects. Other genes of Table 1 are present in a broader range of bacteria, and their expression might depend on regulatory mechanisms different from that of *Y. enterocolitica*. This pathogen is non-motile at body temperature, and a connection between motility and virulence is well-documented [116,117]. For example, a non-motile *flhDC *mutant of *Y. enterocolitica *secretes larger amounts of Yop proteins encoded by the pYV plasmid than the wild-type bacteria [118]. Recently, it was shown that the flagellar master-operon of *X. nematophila *regulates the expression of a novel hemolysin which is required for full virulence of *X. nematophila *against insects [119]. We therefore speculate that motility essentially contributes to the control of the *Y. enterocolitica *switch between two pathogenicity phases towards mammalians and invertebrates [120]. In evolutionary terms, environmental temperature, but not 37°C, appears as the ancient signal for the expression of many genes involved in pathogenicity, confirming the idea that the biological function of many virulence factors has been evolved during the association of bacteria with poikilothermic organisms (see below).

### Evolution of pathogenicity

It has been suggested that bacteria-invertebrate interactions do not only play a role in the transmission of human pathogens but have also shaped their evolution [79]. We identified several common loci representing ancestral clusters of genes important in *Y. enterocolitica *and *P. luminescens *pathogenesis that might have evolved during the association of bacteria with invertebrates, the so-called "pre-vertebrate" pathosphere [121] and then been adapted to more recent pathologies in mammalians. Examples are yersiniabactin, quorum sensing-like regulators, or the urease operon. The complexity of this evolutionary concept is also demonstrated by the fact that the immune systems both of invertebrates and vertebrates are based on phagocytic cells that are attacked by hemolysins, T3SS effector proteins, and many other toxins described above. Thus, it can not be excluded that these virulence factors are able to act on both immune systems [121]. The fact that *P. asymbiotica *has been found to be pathogenic against humans [14] strengthens this hypothesis. Thus, *P. asymbiotica *might be an evolutionary link that is evolving from an insect to a mammal-pathogen. Another example that might enlighten the evolution of bacterial pathogenicity is the plasticity zone (PZ) of *Y. enterocolitica*, a 199 kb key locus for high pathogenicity that includes YAPI_Ye_, secretion systems, hydrogenase loci essential for gut colonization of *S. typhimurium *and *H. pylori *[122], and iron acquisition systems. A second flagellar cluster, Flag-2, is also located within the PZ of *Y. enterocolitica *biotypes 2–5 [120]. It is assumed that the PZ has not been acquired by a single event of gene transfer, but through a series of independent insertions [23]. A comparison of the PZ sequence with the genome of *P. luminescens *is depicted in Fig. 5. A region highly similar to YAPI_Ye _is the genome island *plu0958-1166 *carrying a hemolysin, hypothetical genes, the toxin/antitoxin system CcdA/CcdB, and the type IV pilus. Only few other genes or operons of the PZ are also present in *P. luminescens*, namely a chitinase, two iron acquisition systems, and three *ysa *genes, thus confirming the idea of a patchwork of horizontally acquired genes within the PZ [23]. However, given the extensive transfer of virulence factors between bacteria, the history of pathogen evolution still requires further investigation.

## Conclusion

The comparison of *Y. enterocolitica *and *P. luminescens *at the genomic level performed here provides the database for a better understanding of the genetic basis for their distinct behaviour towards invertebrates and mammals. *Y. enterocolitica *is expected to switch between two pathogenicity phases against insects and mammalians, while the genome of *P. luminescens *must contain the modulators and regulators necessary to change the bacterium from a state of symbiosis with nematodes to pathogenicity against insects, and also from symbiosis-proficient primary variants to symbiosis-deficient secondary variants [17]. Those adaptational processes must be precisely regulated by the bacteria. It was assumed that there are parallels in the regulation of pathogenicity in mammals and insect pathogens [41,123]. However, molecular components of the regulatory networks controlling pathogenicity and mutualism have recently been demonstrated to be very different between *P. luminescens *and *X. nematophilus *with similar life cycles [124]. Dissecting the genomes of *Y. enterocolitica *and *P. luminescens *for putative key regulators, we identified factor groups (AI-2, PAS-4/LuxR like receptors) possibly involved in pathogen-insect association only, those with members contributing to either insect or mammalian pathogenicity (QS, TKS, Usp), and c-di-GMP signalling probably not involved in regulation of activities against insects. Certainly, the question whether fundamental differences in regulatory networks reflect how each of these two bacteria specifically interacts with either the insect or the human host remains to be addressed in more detail.

Bioconversion of its insect hosts is an important stage in the lifecycle of *P. luminescens*. This fact might explain the high number of antibacterial factors directed against possible competitors that are going to colonize the same insect cadaver or that are already present in the insect gut flora [17]. However, no corresponding determinants were identified in the genome of *Y. enterocolitica*. Moreover, *P. luminescens *is pathogenic to a variety of insect larvae, and a dose of <5 colony-forming units directly injected into the blood system is sufficient to kill within 48–72 h [124]. In contrast, only highly concentrated protein extracts of *Y. enterocolitica *are toxic for *M. sexta *[7], and a low insect larvae mortality has been observed following injection of approximately 3.5 × 10^6 ^*Y. enterocolitica *cells into the hemolymph (T. M. Fuchs, unpublished data). This data strongly suggests that *Y. enterocolitica*, similar to *Y. pestis*, has developed a strategy to infect and proliferate in insects, and use these organisms rather as transmission vectors than as pure nutrient source.

Summarizing, *Y. enterocolitica *and *P. luminescens *have evolved partially different and partially similar and therefore probably conserved mechanisms to detect and to react on the insect host. Up to the present time, we are far away from understanding the complexity of bacteria-invertebrate interactions. With the genome comparison carried out here, however, we uncovered several genes which are promising candidate genes involved in insect association and pathogenicity, and therefore created a promising basis for future experimental work.

## Methods

Accession numbers of the genome of *Y. enterocolitica *strain 8081v are AM286415 and AM286416 (plasmid), of the insecticidal toxin genes AJ920332, and of the genome of *P. luminescens *subsp. *laumondii *strain TT01 BX470251. For gene annotation, functional assignments and BLAST analysis, we used the server of the Sanger Institute and the Pasteur Institute. Genome comparison was performed using the ACT software [125], and the GECO comparative genome analysis software [126]. For protein domain analysis, we used the CDART [127], SMART [128] and Pfam [129] algorithms on the NCBI web server. Proteins containing special protein domains were identified by performing a BLAST search of the domain sequence on the genomes of the respective organisms. The threshold for the consideration of protein homologies was a significance value of <10^-04 ^and an identity on the amino acid level of >22% in the BLAST analysis.

## Abbreviations

TCS: two-component system; TPS: two partner secretion; T2SS: type-II secretion system; T3SS: type-III secretion system; T6SS: type-VI secretion system

## Authors' contributions

RH and TMF designed and coordinated the project, carried out the genome analysis, and drafted the manuscript. Both authors have read and approved the final manuscript.
